# Sleep Quality Moderates the Relationship Between Daily Mean Levels and Variability of Positive Affect

**DOI:** 10.1007/s42761-022-00177-8

**Published:** 2023-01-20

**Authors:** Jiyoung Song, Christopher M. Crawford, Aaron J. Fisher

**Affiliations:** 1 Department of Psychology, University of California, Berekely, CA USA; 2 Department of Human Development and Family Studies, Pennsylvania State University, State College, PA USA

**Keywords:** Sleep, Experience sampling, Affect, Affect variability, Affect dynamics

## Abstract

Despite the well-established bidirectional association between sleep and daytime affect, most studies examining this relationship have focused on mean levels of affect. However, research solely focusing on mean levels of affect inherently neglects variability in affect, which has been shown to predict both psychological and physical well-being beyond mean levels. The present study assessed sleep quality and daytime affect using ecological momentary assessment in a combined sample of individuals (*N* = 80; 8,881 observations) with and without anxiety and mood disorders. Results from the present study partially replicated extant work on the negative association between negative affect (NA) variability and subsequent sleep quality. Furthermore, less satisfying sleep amplified the positive relationship between daily mean levels and variability of positive affect (PA). The results did not differ by clinical status. The present study offers novel evidence suggesting that previous night’s sleep quality influences the stability of varying daily levels of PA. Uncovering the dynamics of sleep and affect beyond mean levels will help further elucidate mechanisms linking sleep and subsequent affective experiences.

The bidirectional nature of sleep problems and psychopathology has been well documented (Eidelman et al., 2012). That is, while sleep problems are common symptoms across various mental health conditions, they can also precipitate and perpetuate psychological disorders. One mechanism underlying such a close relationship between sleep problems and psychopathology is the daily interplay of sleep and affect. For example, McCrae et al. (2008) collected sleep diaries and affect data from 103 older adults for 2 weeks and found that greater amounts of wake time during the previous night’s sleep and poorer sleep quality both predicted lower positive affect (PA) and higher negative affect (NA). Consistent with this, de Wild-Hartmann et al. (2013) concluded from a study of 553 women that greater sleep quality during the previous night was significantly associated with higher PA and lower NA, but that only higher PA predicted better subsequent sleep. In a more recent study with a sample comprising 1,165 Dutch participants, Narmandakh et al. (2021) found that while there were significant effects of daytime affect on subsequent sleep quality, these effects were considerably weaker than those of sleep quality on subsequent daytime affect.

Research solely focusing on mean levels of affect, however, inherently neglects other valuable aspects of affective experiences, such as affect variability. Often operationalized as the within-person standard deviation of affect over time, affect variability captures how much the person’s affective experience deviates from their average affect level. Even if an individual’s mean levels of affect are identical over 2 days, their affect variability might differ; the individual might have experienced high fluctuations in affect (high affect variability) one day but low fluctuations in affect (low affect variability) the next day. A growing body of research suggests that both PA and NA variability play an important role in psychological well-being beyond mean levels. Specifically, higher PA variability across 2 weeks has been shown to be associated with worse psychological well-being, lower life satisfaction, and greater anxiety and depression. In fact, higher PA variability within even just a single day can be associated with lower life satisfaction and happiness (Gruber et al., 2013). Researchers have also linked PA variability to physical health. Chan et al. (2016) found that PA variability was positively associated with chest pain, and Jenkins et al. (2018) found a lower antibody response following an influenza vaccination among participants with higher PA variability.

Increased NA variability has also been implicated in various negative mental health outcomes. In a sample of individuals with depressive symptoms, greater NA variability was associated with increased depressive symptoms (Koval et al., 2013). Daily NA variability was also shown to predict future anxiety and depressive symptoms independent of baseline symptom severity and neuroticism (Wichers et al., 2010). Some suggested that NA variability underlies the linkage between neuroticism and increased susceptibility for psychopathology. In a twin study, Jacobs et al. (2011) found a positive association between NA variability and neuroticism and posited that neuroticism might reflect genetic and environmental risks for increased NA variability. In turn, increased NA variability might signal emotion dysregulation and maladaptive heightened emotional reactivity to stressors (Wichers et al., 2015).

Despite evidence supporting the association between affect variability and well-being, most studies examining the relationship between sleep and affect have focused on mean levels of affect. To our knowledge, only one study has directly assessed the relationship between affect variability and subsequent sleep. In Leger et al. (2019), 277 adults aged 65 and older self-reported their sleep behavior every morning and rated their PA and NA three times a day in a 5-to-6-day ecological momentary assessment (EMA) paradigm. Using ordinary least squares regression, the authors found that higher PA variability predicted fewer hours of subsequent sleep independent of mean levels of PA. Though higher NA variability was associated with worse subsequent sleep quality, this effect was no longer significant when adjusting for mean levels of NA. Interestingly, when the authors reanalyzed the data using multilevel modeling to account for the nested structure of data, they did not find significant effects of affect variability on subsequent sleep. Importantly, the authors did not examine how the previous night’s sleep might influence subsequent affect variability despite research demonstrating the bidirectional nature of sleep and affect.

Affect might impact sleep through stress coping and emotion regulatory pathways (Pressman & Cohen, 2005). More specifically, experiences of PA might allow for the accumulation of psychological resources for navigating the stress and hassles of daily life, thereby buffering the deleterious effect of stressors on subsequent health behaviors, such as sleep (Ong et al., 2017). At high mean levels of PA, increased PA variability might therefore inhibit the accrual of the important emotional reserves necessary for successful coping and regulation by decreasing the duration of PA states. Reduced emotional reserves then lead to reduced buffering of stress impacts, ostensibly leading to downstream implications for sleep. This reasoning can be further extended to NA variability, such that periods of increased NA are associated with reduced regulatory capacity (Koval et al., 2013).

Ultimately, affect variation should be examined in concert with mean levels—given the likely impact of floor and ceiling effects on the range and potential variation of affect values. Additionally, mean levels may impact emotion regulation and regulatory goals. For example, at high levels of PA, low affect variability may be more desirable as it indicates the ability to maintain high PA over time (Gruber et al., 2013). The opposite is likely true for NA as high levels of NA with low affect variability indicate persistent NA without downregulation (Koval et al., 2012). Kuppens and Verduyn (2015) described affect variability as an indirect measure of emotion generation and regulation processes. Given that a restful night’s sleep is essential to optimal emotion generation and regulation (Palmer & Alfano, 2017), a poor night’s sleep might lead to difficulties with emotion regulation and consequently experiences of high affect variability at low levels of PA and low affect variability at high levels of NA.

The present study had two primary aims. First, we sought to replicate the extant work demonstrating the effects of affect variability on subsequent sleep. Based on Leger et al. (2019), we hypothesized that higher PA variability would predict shorter duration of subsequent sleep beyond daily levels of PA, and that higher NA variability would predict worse subsequent sleep quality but not beyond daily levels of NA. Second, we sought to examine how previous night’s sleep would influence affect variability. Specifically, we were interested in how the relationship between daily levels of affect and affect variability would vary by previous night’s sleep given that sleep is a daily process and thus needs to be examined at the day level to understand its temporal relations to affective outcomes. We hypothesized that following a shorter and less satisfying previous night’s sleep, higher daily levels of PA would be associated with higher PA variability, and higher daily levels of NA would be associated with lower NA variability.

## Method

### Participants

The present study represents a secondary analysis of previously published data (Fisher et al., 2019). Participants (*N* = 80) were individuals with primary diagnoses of generalized anxiety disorder (GAD; *n* = 23), major depressive disorder (MDD; *n* = 11), both GAD and MDD (*n* = 11), or control participants without any psychiatric diagnoses (*n* = 35). Participant demographics and descriptive statistics are presented in Table 1.

**Table 1:** Participant demographics, sleep, and affect (*N* = 80)

Variable	Without diagnoses (*n* = 35)	MDD only (*n* = 11)	GAD only (*n* = 23)	MDD and GAD (*n* = 11)
	*M (SD)/n* (%)			
Age	28.94 (12.54)	**42.80** (15.80)	33.61 (12.13)	30.55 (13.16)
Race				
Asian/Pacific Islander	9 (25.7)	4 (36.4)	2 (8.7)	3 (27.3)
Black	1 (2.9)	0 (0)	2 (8.7)	1 (9.1)
Hispanic or Latino	5 (14.3)	1 (9.1)	4 (17.4)	1 (9.1)
White	16 (45.7)	5 (45.5)	10 (43.5)	4 (17.4)
Other	4 (11.4)	1 (9.1)	5 (21.7)	2 (8.7)
Sex				
Female	19 (54.3)	6 (54.5)	18 (78.3)	6 (54.5)
Male	16 (45.7)	5 (45.5)	5 (21.7)	5 (45.5)
Sleep				
Duration (hours)	7.45 (0.53)	**6.64** (0.99)	7.17 (0.57)	6.84 (1.03)
Quality (range: 0–100)	82.27 (13.66)	**42.87** (23.24)	**54.10** (17.45)	**49.86** (19.10)
Daily levels of affect				
Positive (range: 0–100)	57.98 (19.92)	**39.35** (8.21)	**40.05** (9.89)	**35.90** (12.45)
Negative (range: 0–100)	12.47(11.20)	**42.61** (16.45)	**39.15** (15.21)	**42.76** (17.33)
Daily affect variability				
Positive	8.68 (4.35)	9.84 (3.65)	10.14 (3.18)	9.52 (3.94)
Negative	4.38 (2.82)	**7.88** (2.42)	**8.72** (2.55)	**8.43** (2.60)

Bolded mean values differ at the *p* = 0.05 level by Tukey’s HSD Test from the non-clinical subgroup. There were no significant differences in mean values between the three clinical subgroups

*MDD* major depressive disorder, *GAD* generalized anxiety disorder

### Measures

#### EMA Survey

For each survey, participants rated their experience of each item over the preceding hours using a 0–100 visual analog slider with the anchors *not at all* and *as much as possible*. Surveys contained the extant symptoms of the Diagnostic and Statistical Manual of Mental Disorders, Fifth Edition criteria for GAD and MDD (*down and depressed*, *hopeless*, *loss of interest or pleasure*, *worthless or guilty*, *worried*, *restless*, *irritable*, *difficulty concentrating*, *muscle tension*, *fatigued*), as well as 11 additional items measuring positive affect (*positive*, *energetic*, *enthusiastic*, *content*), negative affect (*angry*, *afraid*, *anhedonia*), rumination (*dwelled on the past*), behavioral avoidance (*avoided people*, *avoided activities*), and reassurance seeking (*sought reassurance*). The first survey of each day also included items asking about the previous night’s sleep duration and quality. The duration question read “how many hours did you sleep last night?” and was answered in hours between 0 and 24. The quality question read “to what degree did you experience restless or unsatisfying sleep?” and was also rated on a 0–100 visual analog slider. The quality item was later reverse coded, such that a higher score corresponded to a more satisfying sleep.

#### Daily Affect Levels and Variability

We averaged the four items (*positive*, *energetic*, *enthusiastic, content*) for PA (⍺_within_ = 0.80, ⍺_between_ = 0.97; *χ*^2^ = 176.11, *p* < 0.001, CFI = 0.96, SRMR_within_ = 0.036, SRMR_between_ = 0.041) and the seven items (*down and depressed*, *hopeless*, *worthless or guilty*, *irritable*, *angry*, *afraid*, *worried*) for NA (⍺_within_ = 0.82, ⍺_between_ 0 = 0.97; *χ*^2^ = 417.77, *p* < 0.001, CFI = 0.93, SRMR_within_ = 0.048, SRMR_between_ = 0.039) at each observation. We then calculated means and standard deviations of the four daily observations of PA and NA to estimate mean levels and variability of PA and NA for that day, respectively. In our analyses, we only included days with at least three observations, the minimum number needed to calculate affect variability. Thus, the range of daily EMA responses was 3–4 per day.

### Procedure

In the parent study (Fisher et al., 2019), we invited controls and individuals with symptomatic experiences consistent with GAD and MDD to contact the senior author’s laboratory at the University of California, Berkeley. Eligible participants provided intensive repeated measures data via EMA. Participants received a text message to complete survey questions four times per day during their waking hours for 30 days. Surveys were sent using a randomized schedule approximately every 4 hours. That is, the 16 waking hours were divided into four 4-hour blocks, and a survey was randomly sent in each of the 4-hour blocks. There was also an additional constraint that consecutive surveys had to be at least 2 hours apart.

### Analytic Plan

All analyses were conducted in R (Version 4.0.3.; R Core Team, 2020).

#### Multilevel Modeling

To account for the nested structure of our dataset, days within participants, we constructed multilevel models using the *lme4* (Bates et al., 2015) and *lmerTest* (Kuznetsova et al., 2017) packages. All independent variables were person-mean centered before entering them into multilevel models. For our first aim of evaluating the effects of affect variability on sleep, dependent variables were duration and quality of subsequent sleep, and independent variables were PA and NA variability. We then added daily levels of PA and NA as covariates to evaluate whether affect variability was associated with sleep beyond daily levels of affect. For our second aim of examining the effects of previous night’s sleep on the relationship between daily levels of affect and affect variability, dependent variables were PA and NA variability, and independent variables were the sleep duration and quality items, daily levels of PA and NA, and their interactions. We analyzed PA and NA in separate models, and we included random intercepts for participants and used maximum likelihood estimation in all our models. We also included age and sex as covariates to control for their influence on sleep (Luca et al., 2015) and affect variability (Röcke et al., 2009). Additionally, given that our sample consisted of participants with psychiatric diagnoses and healthy controls, we tested for possible three-way interactions between sleep, daily levels of affect, and dummy-coded diagnostic status.

## Results

### EMA Engagement

The total number of non-missing EMA observations was 8,881. Participants were allowed to provide EMA responses past the 30 days. On average, participants completed 111.04 (*SD* = 10.12; range: 90–143) surveys over 33.69 days (*SD* = 4.17; range: 28–55). In the first 30 days, participants completed 100.18 (*SD* = 10.70; range: 64–117) surveys and exhibited a high average compliance rate of 86.3% (*SD* = 9.1%). Their age, sex, race, clinical status, and clinical diagnoses were not associated with their compliance rates, all *p*s > 0.107.

### Affect Variability on Subsequent Sleep

PA variability was not significantly associated with subsequent sleep, both as a sole affect predictor (duration: *B* = 0.00, *SE* = 0.01, *p* = 0.828; quality: *B* = 0.07, *SE* = 0.09, *p* = 0.422) and when covarying daily levels of PA (duration: *B* =  − 0.01, *SE* = 0.01, *p* = 0.825; quality: *B* = 0.06, *SE* = 0.09, *p* = 0.513). Higher daily levels of PA significantly were associated with more satisfying subsequent sleep (*B* = 0.15, *SE* = 0.04, *p* < 0.001), but not duration (*B* = 0.00, *SE* = 0.00, *p* = 0.905). Fixed effect estimates of daily PA mean and variability on subsequent sleep are presented in Table 2.

**Table 2:** Fixed effects of daily positive affect mean and variability on subsequent sleep

Variable	Model 1					Model 2				
	*B*	*SE*	*t*	*p*	*d*	*B*	*SE*	*t*	*p*	*d*
Sleep duration										
Affect variability	0.00	0.01	− 0.22	.828	− 0.01	0.00	0.01	− 0.22	0.825	− 0.01
Mean affect						0.00	0.00	0.12	0.905	0.01
Age	− 0.01	0.01	− 1.51	.136	− 0.34	− 0.01	0.00	− 1.51	0.136	− 0.34
Male sex	0.42	0.17	2.47	.016	0.56	0.42	0.17	2.47	0.016	0.56
Sleep quality										
Affect variability	0.07	0.09	0.80	.422	0.04	0.06	0.09	0.65	0.513	0.03
Mean affect						0.15	0.04	3.48	< 0.001	0.16
Age	− 0.38	0.19	− 1.93	.057	− 0.43	− 0.38	0.19	− 1.94	0.056	− 0.44
Male sex	9.24	5.33	1.73	.087	0.39	9.24	5.33	1.73	0.087	0.39

*d* = Cohen’s *d*

NA variability was not associated with subsequent sleep duration, both as a sole affect predictor (*B* = 0.00, *SE* = 0.01, *p* = 0.509) and when covarying daily levels of NA (*B* = 0.00, *SE* = 0.01, *p* = 0.788). Higher NA variability was significantly associated with less satisfying subsequent sleep (*B* =  − 0.23, *SE* = 0.09, *p* = 0.012), even when covarying daily levels of NA (*B* =  − 0.20, *SE* = 0.10, *p* = 0.038). Higher daily levels of NA significantly did not predict subsequent sleep duration (*B* = 0.00, *SE* = 0.00, *p* = 0.242) and quality (*B* =  − 0.06, *SE* = 0.05, *p* = 0.289). Fixed effect estimates of NA mean and variability on subsequent sleep are presented in Table 3.

**Table 3:** Fixed effects of daily negative affect mean and variability on subsequent sleep

Variable	Model 1					Model 2				
	*B*	*SE*	*t*	*p*	*d*	*B*	*SE*	*t*	*p*	*d*
Sleep duration										
Affect variability	0.00	0.01	0.66	0.509	0.03	0.00	0.01	0.27	0.788	0.01
Mean affect						0.00	0.00	1.17	0.242	0.05
Age	− 0.01	0.01	− 1.51	0.136	− 0.34	− 0.01	0.01	− 1.51	0.136	− 0.34
Male sex	0.42	0.17	2.48	0.015	0.56	0.42	0.17	2.47	0.016	0.56
Sleep quality										
Affect variability	− 0.23	0.09	− 2.53	0.012	− 0.12	− 0.20	0.10	− 2.08	0.038	− 0.10
Mean affect						− 0.06	0.05	− 1.06	0.289	− 0.05
Age	− 0.37	0.19	− 1.93	0.057	− 0.43	− 0.37	0.19	− 1.93	0.057	− 0.43
Male sex	9.21	5.33	1.73	0.088	0.39	9.22	5.33	1.73	0.088	0.39

*d* = Cohen’s *d*

### Previous Night’s Sleep on Affect Variability

**Fig. 1:**
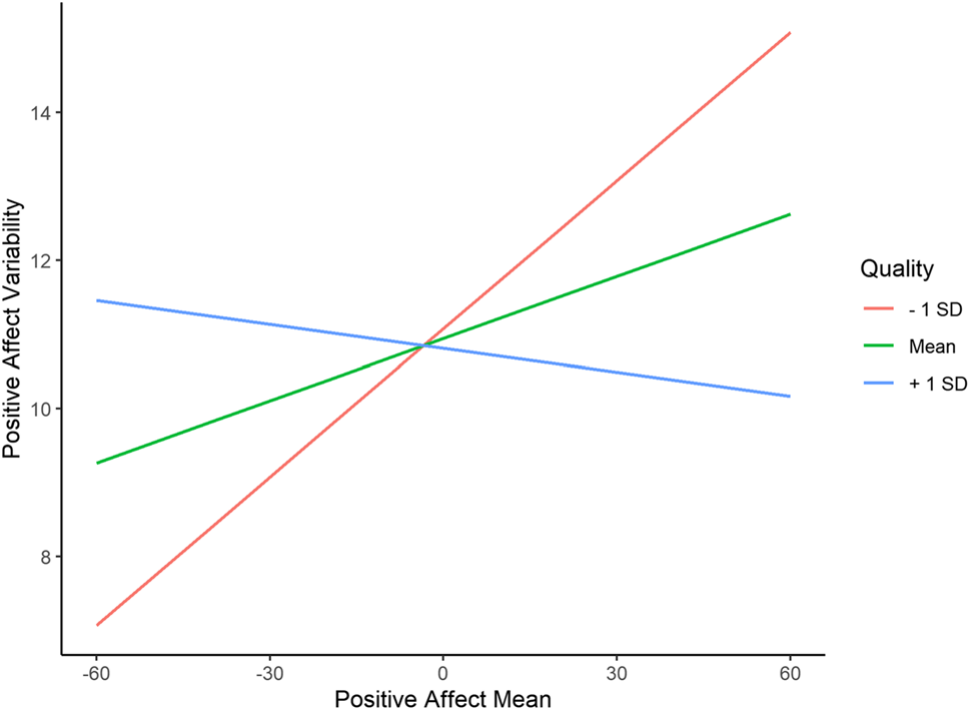
Interaction between sleep quality and daily levels of positive affect on positive affect variability. Both axes are person-mean centered

Previous night’s sleep was not significantly associated with PA variability (duration: *B* =  − 0.03, *SE* = 0.08 *p* = 0.745; quality: *B* =  − 0.01, *SE* = 0.01, *p* = 0.267). The interaction effect between sleep duration and mean levels of PA was not significantly associated with PA variability (*B* = 0.01, *SE* = 0.01, *p* = 0.481). Higher daily levels of PA were associated with lower PA variability following a more satisfying sleep and higher PA variability following a less satisfying sleep (*B* = 0.00, *SE* = 0.00, *p* < 0.001; Fig. 1). Additionally, more satisfying sleep, but not longer sleep, was associated with higher daily levels of PA (duration: *B* =  − 0.01, *SE* = 0.16, *p* = 0.974; quality: *B* = 0.09, *SE* = 0.01, *p* < 0.001) and lower daily levels of NA (duration: *B* = 0.19, *SE* = 0.15, *p* = 0.215; quality: *B* =  − 0.08, *SE* = 0.01, p < 0.001). Fixed effect estimates of previous night’s sleep and daily levels of PA on PA variability are presented in Table 4.

**Table 4:** Fixed Effects of previous night’s sleep and daily mean positive affect on positive affect variability

Variable	*B*	*SE*	*t*	*p*	*d*
Sleep duration					
Sleep duration	− 0.03	0.08	− 0.33	0.745	− 0.01
Mean positive affect	0.03	0.01	2.68	0.007	0.12
Age	− 0.10	0.03	− 3.31	0.001	− 0.75
Male sex	− 1.87	0.87	− 2.15	0.034	− 0.48
Sleep duration $\times$ Mean positive affect	0.01	0.01	0.70	0.481	0.03
Sleep quality					
Sleep quality	− 0.01	0.01	− 1.11	0.267	− 0.05
Mean positive affect	0.03	0.01	2.43	0.015	0.11
Age	− 0.10	0.03	− 3.27	0.002	− 0.74
Male sex	− 1.91	0.88	− 2.18	0.033	− 0.49
Sleep quality $\times$ Mean positive affect	0.00	0.00	− 4.08	< 0.001	− 0.18

*d* = Cohen’s *d*

Previous night’s sleep was not significantly associated with NA variability (duration: *B* =  − 0.03, *SE* = 0.08, *p* = 0.652; quality: *B* =  − 0.01, *SE* = 0.01, *p* = 0.321). Both interaction effects between the sleep items and daily levels of NA were not significantly associated with NA variability (duration: *B* = 0.01, *SE* = 0.01, *p* = 0.077; quality: *B* = 0.00, *SE* = 0.00, *p* = 0.632). Fixed effect estimates of previous night’s sleep and daily levels of NA on NA variability are presented in Table 5.

**Table 5:** Fixed effects of previous night’s sleep and daily mean negative affect on negative affect variability

Variable	*B*	*SE*	*t*	*p*	*d*
Sleep duration					
Sleep duration	− 0.03	0.08	− 0.45	0.652	− 0.02
Mean negative affect	0.15	0.01	13.18	< 0.001	0.59
Age	− 0.04	0.03	− 1.53	0.129	− 0.35
Male sex	− 0.75	0.79	− 0.94	0.350	− 0.21
Sleep duration $\times$ Mean negative affect	0.01	0.01	1.77	0.077	0.08
Sleep quality					
Sleep quality	− 0.01	0.01	− 0.99	0.321	− 0.04
Mean negative affect	0.15	0.01	12.73	< 0.001	0.57
Age	− 0.05	0.03	− 1.56	0.123	− 0.35
Male sex	− 0.72	0.80	− 0.91	0.366	− 0.20
Sleep quality $\times$ Mean negative affect	0.00	0.00	0.48	0.632	0.02

*d* = Cohen’s *d*

Male sex consistently was associated with longer sleep duration (all *p*s < 0.016), and older age was consistently associated with lower variability in PA (all *p*s < 0.002). None of the three-way interaction effects between sleep, daily levels of affect, and diagnostic status on affect variability were significant (all *p*s > 0.267).

## Discussion

The present study evaluated the effects of daytime affect variability on subsequent sleep and the degree to which the previous night’s sleep moderated the relationship between daily levels of affect and affect variability. Psychologically healthy controls and individuals with diagnoses of MDD, GAD, or both completed EMA surveys four times per day for at least 30 days. In the first survey of each day, participants reported previous night’s sleep duration and quality. Within each EMA survey response, we averaged select positive and negative emotion items to estimate PA and NA. We then calculated mean and standard deviation of the daily observations, the latter representing our operational definition of respective affect variability. Finally, we constructed multilevel models that accounted for the nested structure of our data to help answer our research questions.

The current results partially replicate those of Leger et al. (2019). Although we also did not find significant associations between PA variability and subsequent sleep, higher NA variability was associated with worse sleep quality beyond daily levels of NA. One explanation for the divergent findings is that the present study had a considerably longer EMA period (i.e., 30 days vs. 5 days) and was better powered to capture within-person effects. Nonetheless, the association between higher daily NA variability and worse health outcomes is consistent with the literature. Given that emotion dysregulation has been shown to predict sleep disturbance (Grove et al., 2017; Semplonius & Willoughby, 2018), our results might represent emotion dysregulation beyond the mean levels of NA and its impact on sleep quality.

Neither previous night’s sleep duration nor quality impacted subsequent PA or NA variability. For PA variability, however, the effect of daily levels of PA varied as a function of previous night’s sleep quality. Shorter sleep and less satisfying sleep amplified the relationship between daily levels of PA and PA variability such that there were larger differences in the degree of fluctuations in PA between high and low levels of PA. On the other hand, more satisfying sleep reversed the positive association between daily levels of PA and PA variability, meaning when a person experienced high daily levels of PA, well-rested from their previous night’s sleep, they experienced greater stability and lower variability in their PA. This suggests that when individuals experience higher daily levels of PA than is typical, a better-quality sleep the previous night helps to stabilize said levels, thereby enabling the accrual of beneficial psychological resources associated with PA (Ong et al., 2017).

Furthermore, previous night’s sleep quality, but not duration, was predictive of daily levels of PA and NA. It is possible that for mean levels of psychological well-being, how rested an individual feels plays a bigger role than the number of hours they slept (Pilcher et al., 1997; Shen et al., 2018). Another explanation is that a single sleep quality questionnaire captured more dimensions of sleep, such as latency and fragmentation, than a duration questionnaire and thus exhibited greater predictive power for daily levels of affect.

Researchers have consistently linked low-quality sleep with insufficient duration to lower PA and higher NA (Norlander et al., 2005; Steptoe et al., 2008), with similar results in both lab-based (Gordon & Chen, 2014) and real-world settings (Zohar et al., 2005). Furthermore, poorer sleep quality was associated with lowered ability to utilize cognitive reappraisal to downregulate emotions during sad movie clips (Mauss et al., 2013). Putting these findings together, after an unrestful night’s sleep, higher fluctuations in PA coinciding with high daily levels of PA in our study may represent failure to maintain positive emotions.

### Limitations

Although the present study is the first to our knowledge to evaluate the moderating effects of previous night’s sleep on daily levels of affect and affect variability, some limitations should be noted. First, sleep is a complex, multifaceted construct (Harvey & Buysse, 2017), and our two single items broadly assessing the previous night’s sleep may not have fully captured it. Replication studies employing more detailed sleep questionnaires (e.g., the Pittsburgh Sleep Quality Index; Buysse et al., 1989) and objective measures like actigraphy are warranted. Second, the sampling frequency in the present study is lower than the hourly interval recommended for capturing variability in the literature (Dejonckheere & Mestdagh, 2021). Still, affect variability is less susceptible to sampling frequency than other affect dynamics measures like inertia (autoregression) and instability (mean square of successive differences) because it is not sequentially dependent. Future studies with greater sampling frequency should reexamine our research questions to confirm our findings.

## Conclusion

The present study adds findings on the effects of affect variability on subsequent sleep. More importantly, it offers novel evidence suggesting that previous night’s sleep moderates the relationship between daily levels of affect and affect variability. Understanding the dynamics of sleep and affect beyond mean levels will help further uncover mechanisms linking the two.

## Data Availability

The data analyzed for the present study are available in the Open Science Forum repository, https://osf.io/dvcgy/.

## Additional Information

### Data and Code Availability

The datasets generated during and/or analyzed during the current study are available in the Open Science Forum repository, https://osf.io/dvcgy/.

## References

[CR1] Bates, D., Mächler, M., Bolker, B., & Walker, S. (2015). Fitting linear mixed-effects models using lme4. *Journal of Statistical Software*, *67*(1), 1–48. 10.18637/jss.v067.i01

[CR2] Buysse DJ, Reynolds CF, Monk TH, Berman SR, Kupfer DJ (1989). The Pittsburgh Sleep Quality Index: A new instrument for psychiatric practice and research. Psychiatry Research.

[CR3] Chan DKC, Zhang X, Fung HH, Hagger MS (2016). Affect, affective variability, and physical health: Results from a population-based investigation in China. International Journal of Behavioral Medicine.

[CR4] de Wild-Hartmann J, Wichers M, van Bemmel A, Derom C, Thiery E, Jacobs N, van Os J, Simons C (2013). Day-to-day associations between subjective sleep and affect in regard to future depression in a female population-based sample. British Journal of Psychiatry.

[CR5] Dejonckheere E, Mestdagh M, Waugh CE, Kuppens P (2021). On the signal-to-noise ratio in real-life emotional time series. Affect dynamics.

[CR6] Eidelman, P., Gershon, A., McGlinchey, E., & Harvey, A. G. (2012). Sleep and psychopathology. In C. M. Morin, & C. A. Espie (Eds.), *The Oxford handbook of sleep and sleep disorders* (pp. 172–189). Oxford University Press. 10.1093/oxfordhb/9780195376203.013.0010

[CR7] Fisher AJ, Bosley HG, Fernandez KC, Reeves JW, Soyster PD, Diamond AE, Barkin J (2019). Open trial of a personalized modular treatment for mood and anxiety. Behaviour Research and Therapy.

[CR8] Gordon AM, Chen S (2014). The role of sleep in interpersonal conflict: Do sleepless nights mean worse fights?. Social Psychological and Personality Science.

[CR9] Grove JL, Smith TW, Crowell SE, Ellis JH (2017). Preliminary evidence for emotion dysregulation as a mechanism underlying poor sleep quality in borderline personality disorder. Journal of Personality Disorders.

[CR10] Gruber J, Kogan A, Quoidbach J, Mauss IB (2013). Happiness is best kept stable: Positive emotion variability is associated with poorer psychological health. Emotion.

[CR11] Harvey, A. G., & Buysse, D. J. (2017). *Treating sleep problems: A transdiagnostic approach*. Guilford Publications

[CR12] Jacobs N, van Os J, Derom C, Thiery E, Delespaul P, Wichers M (2011). Neuroticism explained? From a non-informative vulnerability marker to informative person-context interactions in the realm of daily life. The British Journal of Clinical Psychology.

[CR13] Jenkins BN, Hunter JF, Cross MP, Acevedo AM, Pressman SD (2018). When is affect variability bad for health? The association between affect variability and immune response to the influenza vaccination. Journal of Psychosomatic Research.

[CR14] Koval, P., Kuppens, P., Allen, N. B., & Sheeber, L. (2012). Getting stuck in depression: The roles of rumination and emotional inertia. *Cognition & Emotion*, *26*(8), 1412–1427. 10.1080/02699931.2012.667392 10.1080/02699931.2012.667392 22671768

[CR15] Koval P, Pe ML, Meers K, Kuppens P (2013). Affect dynamics in relation to depressive symptoms: Variable, unstable or inert?. Emotion.

[CR16] Kuppens P, Verduyn P (2015). Looking at emotion regulation through the window of emotion dynamics. Psychological Inquiry.

[CR17] Kuznetsova, A., Brockhoff, P. B., & Christensen, R. H. B. (2017). lmerTest package: Tests in linear mixed effects models. *Journal of Statistical Software*, *82*(1), 1–26. 10.18637/jss.v082.i13

[CR18] Leger KA, Charles ST, Fingerman KL (2019). Affect variability and sleep: Emotional ups and downs are related to a poorer night’s rest. Journal of Psychosomatic Research.

[CR19] Luca, G., Haba Rubio, J., Andries, D., Tobback, N., Vollenweider, P., Waeber, G., ... & Tafti, M. (2015). Age and gender variations of sleep in subjects without sleep disorders. *Annals of Medicine*, *47*(6), 482-491.10.3109/07853890.2015.1074271 10.3109/07853890.2015.1074271 26224201

[CR20] Mauss IB, Troy AS, LeBourgeois MK (2013). Poorer sleep quality is associated with lower emotion-regulation ability in a laboratory paradigm. Cognition & Emotion.

[CR21] McCrae CS, McNamara JPH, Rowe MA, Dzierzewski JM, Dirk J, Marsiske M, Craggs JG (2008). Sleep and affect in older adults: Using multilevel modeling to examine daily associations. Journal of Sleep Research.

[CR22] Narmandakh A, Oldehinkel AJ, Masselink M, de Jonge P, Roest AM (2021). Affect, worry, and sleep: Between- and within-subject associations in a diary study. Journal of Affective Disorders Reports.

[CR23] Norlander T, Johansson Å, Bood SÅ (2005). The affective personality: Its relation to quality of sleep, well-being and stress. Social Behavior and Personality: An International Journal.

[CR24] Ong AD, Kim S, Young S, Steptoe A (2017). Positive affect and sleep: A systematic review. Sleep Medicine Reviews.

[CR25] Palmer CA, Alfano CA (2017). Sleep and emotion regulation: An organizing, integrative review. Sleep Medicine Reviews.

[CR26] Pressman SD, Cohen S (2005). Does positive affect influence health?. Psychological Bulletin.

[CR27] Pilcher JJ, Ginter DR, Sadowsky B (1997). Sleep quality versus sleep quantity: Relationships between sleep and measures of health, well-being and sleepiness in college students. Journal of Psychosomatic Research.

[CR28] R Core Team (2020). *R: A language and environment for statistical computing* [Computer software]. R Foundation for Statistical Computing. https://www.R-project.org/.

[CR29] Röcke C, Li SC, Smith J (2009). Intraindividual variability in positive and negative affect over 45 days: Do older adults fluctuate less than young adults?. Psychology and Aging.

[CR30] Semplonius, T., & Willoughby, T. (2018). Psychosocial adjustment throughout university: A longitudinal investigation of the roles of sleep quality and emotion dysregulation. *Journal of Youth and Adolescence*, *47*(6), 1267–1278. 10.1007/s10964-018-0826-5 10.1007/s10964-018-0826-5 29476457

[CR31] Shen L, van Schie J, Ditchburn G, Brook L, Bei B (2018). Positive and negative emotions: Differential associations with sleep duration and quality in adolescents. Journal of Youth and Adolescence.

[CR32] Steptoe A, O’Donnell K, Marmot M, Wardle J (2008). Positive affect, psychological well-being, and good sleep. Journal of Psychosomatic Research.

[CR33] Wichers M, Peeters F, Geschwind N, Jacobs N, Simons CJP, Derom C, Thiery E, Delespaul PH, van Os J (2010). Unveiling patterns of affective responses in daily life may improve outcome prediction in depression: A momentary assessment study. Journal of Affective Disorders.

[CR34] Wichers M, Wigman JTW, Myin-Germeys I (2015). Micro-level affect dynamics in psychopathology viewed from complex dynamical system theory. Emotion Review.

[CR35] Zohar D, Tzischinsky O, Epstein R, Lavie P (2005). The effects of sleep loss on medical residents’ emotional reactions to work events: A cognitive-energy model. Sleep.

